# Work process of nursing professors ^1^

**DOI:** 10.1590/1518-8345.1941.2946

**Published:** 2017-12-04

**Authors:** Denisse Parra Giordano, Vanda Elisa Andres Felli

**Affiliations:** 2 Doctoral student, Escola de Enfermagem, Universidade de São Paulo, São Paulo, SP, Brazil. Assistant Professor, Departamento de Enfermería, Universidad de Chile, Santiago, Stgo, Chile.; 3 PhD, Associate Professor, Escola de Enfermagem, Universidade de São Paulo, São Paulo, SP, Brazil.

**Keywords:** Occupational Health, Quality of Life, Faculty, Nursing, Working Conditions, Work

## Abstract

**Objective:**

to analyze the work process of nursing professors.

**Method:**

descriptive, exploratory and qualitative study, developed with a focus on critical epidemiology, carried out at a School of Nursing in Chile. The research subjects consist of 17 nursing professors, with whom individual semi-structured interviews were carried out and nine participated in a focus group. The Ethics Committee approved this study.

**Results:**

88.2% were female, mean age of 42 years, 47% were married, 94% were Chilean, average length of service in the institution of 2.8 years, and 23.5% had a master’s degree. Regarding the work process, the students were the work object, the tools used were the knowledge and the experience as a nurse, and the work environment was considered good. Regarding the form of work organization, 76% have a 44-hour workweek, the wage was considered inadequate and the workload was higher than foreseen in the contract. The dialectic of the nursing work process is evidenced, demonstrating the contradiction between the low wages and labor overload and the narratives reporting a good work environment, personal fulfillment and transcendence that goes far beyond work.

**Conclusions:**

the work process allows describing the work components of the nursing professors, which are consistent with the results of the literature and show the dialectic of the nursing work process.

## Introduction

Nursing is known as an activity since the beginning of humanity, because there have always been people in need of health care, and care is understood as the essence of this profession^1^ ^-^ ^2^, which evolved from an occupation without formal preparation to a profession with training at higher education level. It can be developed in different areas, such as administration, medical care, education, research and political participation, requiring a set of specific skills and abilities for each of them^3^. The nursing profession is inserted as a social practice in the world of work and is marked by historical, social, economic and political events^3^ ^-^ ^4^. In the course of the nursing development process, world has been undergoing significant changes due to globalization, and today, a capitalist mode of production has caused many changes in the Work Process, which are also observed in the nursing work. This has also meant changes in the profile of work and workers, which had to get adapted to the new technologies and the current economic model^4^. Therefore, there is not only a need for professionals trained in the area of knowledge, but also competent professionals with specific skills. In this fashion, there is a need for professors with competencies in accordance with this new reality, as well as significant changes in the teacher-student relationships^4^ ^-^ ^6^. In Latin America, Chilean society was also impacted by globalization, reflected in the rapid growth of the population, changes in its structure, changes in the morbidity and mortality profile, increase in life expectancy and improvements in living and working conditions, such as food, wage, etc.^7^. 

Because of the change in the Chilean economic model, there was an increase in the university offer as a business possibility, generating an increase in the offer of nursing training^7^, which, in turn became the most sought-after career at the national level. In this context, there has been a rise in the number of nursing professionals who develop their work process in the teaching area. The scenario in nursing teaching makes the work process particularly complex, because training in nursing requires a specific environment, and, in addition, in the teacher-student relationship there is also the patient^8^.

Work is a fundamental condition that sets out the position of individuals in society and defines the power of consumption and access to decent living conditions^9^, representing more than just the relationship with employment or wages, but a form of social insertion^8^. In the health area, work is characterized by the provision of services, consisting of provision of health care to individuals, and thus, being placed in the tertiary sector of the countries’ economies. Understanding nursing as a career requires the understanding of the health practices as social practices, which transcends the professional and technical magnitude^3^. At the same time, it is a dynamic practice that requires a constant review of its components in the face of the changes in society and health care models^10^. Specifically in the teaching Work Process, the professional relationships are differentiated and the teaching activities are performed in practice and, therefore, there is a peculiarity in the way of experiencing the health-disease process^8^ ^,^ ^11^. 

The Work Process consists of three elements: work object, work instruments and the work itself^3^ ^,^ ^9^. Work object is where the activity being carried out will reach its effect and that it will be transformed during the process to become a product^3^ ^,^ ^9^, so when this transformation process occurs in a person, as in the case of Education, such a person is called subject of work^3^. The work instrument is a set of tools that are in between the work itself and the work object, serving as a means of its action on it^3^ ^,^ ^9^; and so the nursing professor transforms the work object (subject) using a specific knowledge and appropriate tools^3^. The activity refers to the work and the specific organization of the work for a specific purpose^3^ ^-^ ^4^ ^,^ ^9^, as well as to working conditions, such as working hours, wage, institution’s schedule, hierarchy, among others^8^. 

In order to understand work as a process, not only as a risk factor to people’s lives, an approach from a critical epidemiology point of view is necessary, considering that epidemiological processes are active and defined in the social reproduction of each group^10^. 

Thus, according to the point of view of Naomar Almeida Filho, the main promoter of the critical epidemiology in Brazil, it can be studied from both a qualitative and quantitative perspectives, as long as its historical and social context is maintained^12^.

The changes in the labor market and the increase in the number of nursing professionals dedicated to teaching in Chile have contributed to the growth of the role of nursing in the teaching-learning process, as well as other areas of its competence. All of this imposes the need to analyze the Work Process of nursing professors of a public university, which is the objective of the present work.

## Method

It is a descriptive, exploratory and qualitative study, developed with a focus on critical epidemiology. The study scenario is the Nursing School of the University of Chile, founded in 1906, which grants the title and the bachelor’s degree in Nursing and has been accredited for seven years (maximum grantable). In addition, it belongs to a public university that stands out as a reference at national and international level. The study population was composed of all nursing professors, totaling 52 people, by applying the following selection criteria: 22, 33 or 44-hour workweek, work as a nursing professor for six months or more. People on vacation or medical leave were excluded from the study, resulting in 25 potential participating subjects. Individual interviews coordinated by the main researcher, who had a prior work relationship with the participants, were carried out with 17 nursing professors, through the application of a questionnaire on sociodemographic data and the guiding question “How is your work process as a nursing professor?”, which allowed the phenomenon to be understood by researchers. Participants agreed to participate in the study (there were no withdrawals or refusals to participate in the interviews) in a room at the School of Nursing with total privacy, lasting between 15 and 32 minutes, in April 2014. In order to guide the analysis process, interviews were recorded and transcribed in full. The data were subjected to content analysis, considering predefined categories according to the Work Process, object, instruments used, environment and form of organization. For data presentation, the interviewees were identified using the letter E, and their numbers were specified. The study was carried out according to the ethical requirements, being approved by the Human Research Ethics Committee of the Faculty of Medicine of the University of Chile, on January 21, 2014. The school administration also authorized the study, and all subjects signed the Informed Consent Form. 

## Results

In total, 17 nurses were interviewed. Regarding the sociodemographic characterization of the population, the study shows that 88% of the subjects are female, mostly young adults, 47% are married, 64.7% have children and 94% are Chilean. 

Regarding the staff training, 100% of professors have postgraduate qualification (*lato sensu*) of about 200 hours. Ten professors are currently enrolled in a Master’s program, three already have a Master’s degree and four have not yet started their postgraduate studies.

Regarding the work history, professors interviewed in the School of Nursing have, on average, 2.8 years of service in the University, most of the participants work in the institution between three and six years, as shown in Table 3. Workers have weekly working hours, so that 76.5% have a 44-hour workweek, 17.6% have 33-hour workweek and 5.9% have a 22-hour workweek. With regard to the type of contract of the professors, 52.9% take up the academic career within the academic sector and 47.1% are engaged in the academic career as part of the collaborative team. The second type of contract aims at protecting the remuneration of the nursing professors, due to the lack of qualification on entering the academic career. According to the academic hierarchy, 94.1% are classified as Instructors and 5.9% as Assistant Professors, with these being the two hierarchies present in the School of Nursing under study. In addition, the interviewed nursing professors work in the four areas of the School: 29.4% in the Community Health, 29.4% in Hospital Pediatrics, 29.4% in Adult Hospital and 11.8% in Mental Health. It is important to mention that 100% of the interviewees are responsible for monitoring or supervising students in clinical practice.

**Table 1:** Sociodemographic characterization of nursing professors (n=17), according to sex, age, marital status, number of children and nationality. Santiago, Chile, 2014

**Characteristic**		**Number**	**Percentage (%)**
**Sex**			
	**Female**	**15**	**88**
	**Male**	**2**	**12**
**Age**			
	**Young adult**	**10**	**58.8**
	**Middle-aged adult**	**4**	**23.5**
	**Older adult**	**3**	**17.6**
	**Average**		**44.2**
**Marital status**			
	**Married**	**8**	**47**
	**Single**	**5**	**29**
	**Divorced**	**2**	**12**
	**Separated**	**2**	**12**
**Have children**		**11**	**64.1**
**Nationality**			
	**Chilean**	**16**	**94**
	**Foreigner**	**1**	**6**

**Table 2:** Postgraduate qualification of nursing professors (n=17), according to specialization, master’s and doctorate degree. Santiago, Chile, 2014

**Post-graduation Level**		**Number**	**Percentage (%)**
**Specialization**		**17**	**100.0**
**Master’s degree**			
	**Studying**	**10**	**58.8**
	**Completed**	**3**	**17.6**
**Doctorate degree**		**0**	**0**
**Without postgraduate qualification**		**4**	**23.5**

**Table 3:** Work history of nursing professors in the School of Nursing (n=17), according to length of service, weekly workload, academic hierarchy, type of career, work area, practical experience. Santiago, Chile, 2014

**Characteristic**		**Number**	**Percentage (%)**
**Length of service (in years)**			
	**Mean**	**17**	**2.8**
	**1**	**6**	**35.2**
	**2**	**1**	**5.9**
	**3**	**3**	**17.6**
	**4**	**6**	**35.2**
	**8**	**1**	**5.9**
**Weekly workload (in hours)**			
	**44**	**13**	**76.5**
	**33**	**3**	**17.6**
	**22**	**1**	**5.9**
**Academic hierarchy**			
	**Instructor**	**16**	**94.1**
	**Assistant Professor**	**1**	**5.9**
**Type of career**			
	**Academic**	**9**	**52.9**
	**Professional**	**8**	**47.1**
**Work area**			
	**Community health**	**5**	**29.4**
	**Hospital pediatrics**	**5**	**29.4**
	**Adult hospital**	**5**	**29.4**
	**Mental health**	**2**	**11.8**
**Practical experience**		**17**	**100%**

For the analysis of the Work Process, its elements - object, means/instruments and forms of organization and divisions - were abstracted from the subjects’ discourses; which were present since the Individual Interview.

Regarding the work object, the mentioned sentences show that all professors consider that their work object is focused on the biopsychic aspect of students, who experience the teaching-learning process, transforming knowledge. The first topic that emerged in relation to the work object is the training of new professionals: *the students acquire tools that allow them to develop skills and strengths for their professional development* (E13), *contributing to the training of the new professionals* (E3); it directly shows the teachers’ focus on the students as their raw material, to whom the work is applied, in order to adapt the new nursing professional to the institutional context. Likewise, the topic of Personal Realization was identified in relation to training: *it has to do with professional development, because in order to do this, you also need to have some requirements as a way to enhance your own professional development, I mean, your knowledge levels and things like that* (E5); in this way, at the same time that the work transforms an object, it also transforms the man who performs it^9^, the teachers refer to a personal modification being developed in this area of their profession, in addition to the change caused in the student. 

The second element are the resources used in the teaching work, which can be separated into two areas, with the first corresponding to the workforce, that is, the worker’s own abilities in terms of agility, competence or skills^9^. This study evidences the knowledge and experience as a nurse in preparing the classes, tutoring and practical training, especially in terms of specific professional training related to the teaching area. Knowledge and experience: *on the one hand, there is the practical experience, but there is also the theoretical training provided, so that both can complement each other* (E8). The other instruments refer to the infrastructure available in the institution, both the classrooms and the facilities to carry out the practical experiences with the students, as shown in the following interview, are considered adequate. Infrastructure: *there are offices where you can do other things, so I believe that the infrastructure is adequate for the activities to be carried out* (E4). 

Regarding the forms of organization, professors mention them in their speeches in several respects. The nursing teaching work is developed through a certain form of organization, such as a work schedule that runs from eight in the morning to five in the afternoon on working days. Work schedule: *I have a thirty-three hour workweek from eight in the morning to five in the afternoon* (E15) during the working day, the nursing professors carry out activities related to teaching, research, extension, with the first being performed mainly with undergraduate students. Likewise, it is emphasized that 100% carry out practical experience monitoring, including the School principal. Activities distribution: *I carry out many practical activities; I come to the practical class and supervise students of different levels, from those who begin their internships to interns. A lot of work is also carried out in the administrative scope, much is done ... issues related to the organization of the disciplines, work teams, practical activities, elaboration of instruments, development of instruments, texts and classes, besides having to teach in class as well* (E4). 

Despite the form of organization of the teaching work, the environment is a key feature, providing sufficient space for the physical and emotional development of the work. In the present study, the environment is considered as good for most subjects. Work Atmosphere: *it is very good, I think that the atmosphere is good, that the interpersonal relationships are good, that although we think differently, or that we have differences, I mean, in relation to our ages and how we think, I think the environment is good, it is pleasant* (E4). The wages are considered inadequate based on two aspects; first, they are not considered as representative of the workload performed, and second, they are low when compared to the current job market. Remuneration and market wage: *here the wage is fair, although, I think it should be paid according to the amount of activities one performs, because it is clearly perceived that there are people who earn the same amount of money as the other ones, and do much less* (E2); *I think it is lower than the market price, if we compare with the other universities, that is, that´s why suddenly private universities offer you much more, with wages considered as exorbitant, i.e., wages of about ... you can earn the same amount working half of normal working hours, and in fact, you can earn much more* *per class* (E5). 

Finally, work transcends to the human being and is recognized as a transforming action of reality, being an experience on itself^9^ and providing an additional purpose to the nursing professor. Transcendence of work: I *always enjoyed teaching, and because I liked it, I have always sought my work, because there is a purpose, because there is transcendence in doing it* (E14). 

## Discussion

### Sociodemographic and labor characteristics

As regards gender, studies in Brazil also show that the nursing workforce is composed mainly of women, 100%^11^ ^)^ and 97.3%^13^, in the United States 78.4%^2^, Colombia 91.6%^14^, China 95.2%^15^ and in Portugal 66.7%^16^. This composition shows the historical insertion of women into society and the Nightingale heritage as a female profession^11^, reproducing a role of nurse and educator^5^. As for the age range, 85%^15^ ^)^ are aged from 20 to 40 years, like in a study carried out in China, but unlike other studies conducted in Brazil, in which most professors were middle-aged adults, 66.7%^11^. This shows that the School’s workforce is mainly young, partly due to the hiring of new staff in 2010, when new professors were hired, as well as the implementation of a competency-based curriculum in 2013, which caused an increase of teaching staff numbers.

Regarding marital status, other studies also report a larger proportion of married professionals, with 86.3%^15^ in China, 79%^13^ ^)^ and 54.8%^11^ ^)^ in Brazil. This suggests that the professors of the University under study begin young in the area of education and, therefore, have not yet married, which is corroborated by the fact that most professors belong to a young adult age group. Regarding children, the data of this study resemble an study carried out in Colombia^14^, which showed that 63.2% of nursing professors had children.

The professional qualification has a key value in the new model of capitalist labor, which requires the development of new competences by the professors. However, this knowledge is not limited to its application, since it requires from the nurses a reflexive exercise, involving skills and learning^4^ ^,^ ^6^. However, nurses recognize the need to invest in the teaching career and maintain the motherhood as a social essence^5^, which would explain why four of the teachers interviewed still did not have postgraduate qualification and are not enrolled in a postgraduate program. It is important to emphasize that some nurses who return to their nursing care activities mention the reasons why they should complete a certain academic degree^2^. Regarding the training at master’s level, the results are consistent with a study carried out in the United States that shows that 53.4% of professors have a master’s degree and 10.8% have a doctorate degree^2^, in Portugal 45.4% have a master’s degree and 18% have a doctorate degree^16^ and in China 63.9% have a master’s degrees^15^. Similarly, in Brazil, 38% have a master’s degree, 23.8% have a doctorate degree and 4.8% have postgraduate qualification^11^. 

As for the labor aspects, the length of service in the institution is not consistent with the results of a study carried out in Colombia that shows that 47.3% have more than five years of service in the institution^14^. Regarding the working arrangement, a study developed by the National Nursing Council (Cofen), Brazil 2016, refers mainly to those paid per hour worked or on a part time scheme, with 65.8% on a full-time schedule and, only 11.1%, with an exclusive dedication type of work^17^. These results are contrary to the findings of the present study; however, it should be taken into account that only people with a workweek of 22 hours or more were interviewed here. This situation hinders the teaching development and the performance of research and extension activities carried out by the nursing professors.

Regarding the type of contract, the reality of the School under study is different from that of Colombia, which has 46.3% of professors with a permanent contract^14^ and Portugal, with 76.5% of contracted professors^16^; since no professor in the studied School of Nursing in Chile works under this labor contract. This is consistent with a mostly young teaching staff and who has a postgraduate qualification, as shown in the results. Dissatisfaction with the academic hierarchy and the absence of a fixed-term contract provokes a situation of instability in the employment relationship, which can cause suffering on the part of the nursing professors^8^, due to their constant concern to advance their academic career.

The university teaching development in nursing involves a multiplicity of tasks, together with the requirements for the development of the teaching-learning process^5^ ^,^ ^8^ ^,^ ^18^, as evidenced in this study. This is reinforced by the direct monitoring in the practical experience of first to fifth year undergraduate students, which is performed by 100% of the interviewees. It is recognized that the professional relationship between professors and the students under their monitoring increases job satisfaction, contributing to a greater permanence of nurses in the teaching career^2^, when compared to other areas of nursing domain that showed job dissatisfaction^19^. 

### Work process

In relation to the Work Object, the main meaning of the nursing teaching work is the contribution provided in the training of students as nurses, with skills and abilities for professional practice, being also able to face the social coexistence and the labor market challenges^4^. This is achieved by recognizing the students as their work object^3^ ^,^ ^6^ and the importance of their actions for the training of students^18^ ^,^ ^20^. Likewise, nursing professors feel responsible for the preparing and learning of students^8^. In another study, healthcare area professors allude to the fact that the teaching practice involves personal and professional fulfillment, as well as welfare and satisfaction through the recognition of their work^21^, that is, the nursing professors refer to the pleasure to perform their job and the recognition of their work by the students^8^. On the other hand, a study carried out in Colombia shows the lack of personal fulfillment demonstrated by most nursing professors^14^.

Regarding the means of work, due to market demands, nurses have to deepen their professional competences^4^ ^,^ ^6^ ^,^ ^22^ and keep up-to-date to answer the students’ questions^6^ ^,^ ^18^ ^,^ ^23^. Professors recognize the exchange of experiences and knowledge provided through the professional relationship with the students^20^ ^-^ ^22^. The nursing professors use their own experiences and existing knowledge to improve their competences as a teacher^4^; and teachers in the healthcare area also refer to the production of knowledge^6^ ^,^ ^20^, which must be legitimized through publications^5^. In the same way, in a study conducted in Chile, students recognize knowledge as the main virtue of a “Good Nursing Professor”^6^. In a Brazilian study^11^, 56.7% of professors mentioned that the workplace physical conditions are adequate, but when the workplace do not meet the requirements to develop teaching, the structure of the institution also falls within the group of elements that cause suffering^8^.

As for Work Organization, most professors also carry out various teaching activities at undergraduate and postgraduate levels, and devote many extra hours to meetings, teaching production, presentation at events, and scientific production through indexed publications^5^. A pleasant atmosphere at work involves the well-being of the workers, giving them pleasure for the work done^8^ ^,^ ^19^. In other realities, the work environment is also considered as a very important factor for the work process development of the healthcare professors and, although there are obstacles in the lives of Brazilian professors, they agree that an environment that promotes quality of life and quality of life at work is also important in many areas^11^. Similarly, the nursing professors also report that the different activities of their jobs may not be considered as health-promoting actions^2^ ^,^ ^21^. In one study, they argue that the teaching environment negatively affects their quality of life^21^. 

Regarding the wage, the nursing professors identify a distress due to the low wage and the poor working conditions^2^ ^,^ ^8^, which contrast with the findings of this study^15^. Similarly, in a study conducted in Medellin, most professors disagree with their salaries^14^. Wages are considered as an important element for job satisfaction, as in other areas of nursing development^2^. This instability mentioned in relation to the workload can cause suffering on the part of the nursing professors^8^. In contrast, most professors are satisfied with their pay and say that there is a balance between wages, as reported in a Brazilian study^11^. However, there is a disparity between the wages of public and private institutions, considering the same proposed work schedule^13^. This requires that professionals have two or more jobs as way to ensure a socially necessary income, which is also mentioned in this study. 

In the historical and social context of work, the social insertion of individuals in the labor market^9^ directly influences the shaping of their identity, bringing feelings of fullness, achievement, recognition and social utility for people^8^, which transcends the consumerism and the acquisition of material goods. This study evidences how the nursing work process is from the point of view of critical epidemiology, demonstrating the dialectic nature experienced by the professors. On the one hand, there are the low wages and the high workload and, on the other hand, there is the personal fulfillment and the occurrence of a transcendence that goes far beyond the work itself as a contribution to personal development.

The main limitation of this study is that it was carried out only in the context of a public university. However, the inclusion of private universities in future research could be considered as a way to complement the present study.

## Conclusions

The scenario of the work process development is complex, because the training in nursing requires a specific environment and, in addition to the teacher-student professional relationship, there is still the patient. The nursing professor develops the teaching process in its entirety, applying it not only to the students, but also to the person, family and community.

The development of this study allowed analyzing the work process reported by the nursing professors in a public university, achieving the proposed general objective. 

This work provided the sociodemographic characterization of nursing professors, and it could be concluded that the nursing professors of the public university under study are mainly middle-aged adult women.

With regard to the Work Process, the nursing professors have their focus mainly on the training of students; the structural settings are adequate to teach classes; most professors have a full weekly workload (44 hours); the work environment is considered as good. However, they reported low wages and high workload, so that they have to allocate time from their personal life to the work.

In conclusion, the Work Process from a perspective of critical epidemiology allows describing in depth the different components of the work process of nursing professors; in addition to showing the dialectic in the development of nursing in the education context.
